# Genomic variants reveal differential evolutionary constraints on human transglutaminases and point towards unrecognized significance of transglutaminase 2

**DOI:** 10.1371/journal.pone.0172189

**Published:** 2017-03-01

**Authors:** Kiruphagaran Thangaraju, Róbert Király, Máté A. Demény, János András Mótyán, Mónika Fuxreiter, László Fésüs

**Affiliations:** 1 Department of Biochemistry and Molecular Biology, Faculty of Medicine, University of Debrecen, Debrecen, Hungary; 2 MTA-DE Momentum Laboratory of Protein Dynamics, Faculty of Medicine, University of Debrecen, Debrecen, Hungary; 3 MTA-DE Stem cell, Apoptosis and Genomics Research Group of Hungarian Academy of Sciences, Faculty of Medicine, University of Debrecen, Debrecen, Hungary; University of Maryland School of Medicine, UNITED STATES

## Abstract

Transglutaminases (TGMs) catalyze Ca^2+^-dependent transamidation of proteins with specified roles in blood clotting (F13a) and in cornification (TGM1, TGM3). The ubiquitous TGM2 has well described enzymatic and non-enzymatic functions but in-spite of numerous studies its physiological function in humans has not been defined. We compared data on non-synonymous single nucleotide variations (nsSNVs) and loss-of-function variants on TGM1-7 and F13a from the Exome aggregation consortium dataset, and used computational and biochemical analysis to reveal the roles of damaging nsSNVs of TGM2. TGM2 and F13a display rarer damaging nsSNV sites than other TGMs and sequence of TGM2, F13a and TGM1 are evolutionary constrained. TGM2 nsSNVs are predicted to destabilize protein structure, influence Ca^2+^ and GTP regulation, and non-enzymatic interactions, but none coincide with conserved functional sites. We have experimentally characterized six TGM2 allelic variants detected so far in homozygous form, out of which only one, p.Arg222Gln, has decreased activities. Published exome sequencing data from various populations have not uncovered individuals with homozygous loss-of-function variants for TGM2, TGM3 and TGM7. Thus it can be concluded that human transglutaminases differ in harboring damaging variants and TGM2 is under purifying selection suggesting that it may have so far not revealed physiological functions.

## Introduction

Transglutaminases (TGM) are a family of Ca^2+^-dependent protein transamidating and cross-linking enzymes implicated in variety of biological processes. So far eight active and one inactive member (F13a, TGM1-TGM7, and Band 4.2) have been identified in humans. F13a plays a pivotal role in blood coagulation by crosslinking fibrin, TGM1 is essential for proper cornification in the skin with contribution from TGM3 and TGM5, and TGM4 seems to have a role in prostate function [1, 2]. The role of TGM6 and TGM7 are not known. The most studied TGM2 is widely expressed exhibiting transamidase, isopeptidase, protein disulphide isomerase, protein kinase, GTP and ATP binding and hydrolyzing activities [1, 2]. Its transamidase activity is reciprocally regulated by GTP and Ca^2+^ binding, which are followed by a large conformational change resulting in either an inactive closed or a potentially active opened conformation, respectively [3]. TGM2 interacts with many other interacting partners including fibronectin and can act as a protein scaffold (reviewed in [4]). TGM2 is involved in a broad range of cellular processes (including transmembrane signaling, mitochondrial functions, gene expression regulation, autophagy, and apoptosis) and has been related to pathological conditions such as inflammation, autoimmune disease, cancer progression, and neurodegenerative disorders (reviewed in [5]). TGM2 knock out mice are born and live without any obvious phenotype—although they have decreased defense to some environmental stress and diseases which develop by age (reviewed in [5, 6]). Understanding the physiological functions of TGM2 in humans is still awaited since no TGM2 deficiency or dominant TGM2 mutant has been observed and systematic inhibitor studies could not be carried out.

After the completion of the human genome project and large scale exome sequencing presented in various databases, it has become evident that vast majority of our proteins are polymorphic to various genomic changes including single nucleotide variations and loss-of-function alterations. Rapid population growth coupled with inefficient and weak natural selection has led to excessive rare variants in our genome, some are associated with severe pathological phenotypes as a result of damaging mutations [7]. They can affect protein function and are implicated in both Mendelian and complex diseases [8]. In general, out of 13,000 exonic variants present per person, about 60% are non-synonymous [9]. Non-synonymous single nucleotide variants (nsSNVs) located in protein cores and protein-protein interfaces are disease-causing [9] owing to protein misfolding or instability or altered protein-protein interactions. Recent whole-genome and exome sequencing data from different populations, including bottlenecked and consanguineous ones, have revealed a large number of genes with homozygous or compound heterozygous loss-of-function (LOF) variants leading to human knockouts and efforts are underway to find phenotypic consequences of these knockouts [10, 11, 12]. In addition, new genome editing technologies made it possible to knock out human protein coding genes one by one and to test which are essential for cell proliferation and survival in culture highlighting approximately 2000 such genes [13, 14, 15].

We noticed earlier in limited exome sequence data sets that the protein coding sequence of human TGM2 has the lowest variability when compared to other members of the transglutaminase family [16]. In the present study we analyzed large scale population genomics data in an effort to understand evolutionary forces that have shaped this family of enzymes in human populations. We have screened the Exome Aggregation Consortium (ExAC) database for single nucleotide variant types in the transglutaminase family and compared the TGM2 variants with other members of the family. We focused on the potential impact of damaging TGM2 nsSNVs on protein stability, secondary structure, functional sites including calcium, fibronectin and GTP binding sites. We also studied intrinsically disordered regions (IDRs) which affect protein dynamics and could interfere with conformational changes as well as harbor short linear motifs (SLiMs) for protein interactions and modifications. To this aim, the evolutionary constraints on TGMs and population frequencies of the homozygous transglutaminase nsSNVs have been compared. The homozygote allele variants with nsSNVs available in public datasets were produced, analyzed and tested in biochemical assays. We have also searched for data to see the prevalence of transglutaminase LOF variants and human knock outs as well as disease causing mutations in different populations to see how essential these proteins are for humans.

## Materials and methods

All materials were purchased from Sigma (St Louis, MO, USA) unless otherwise indicated. The FLpepT26 peptide was obtained as published in Ref. [17] and S100A4 (GST) as published in Ref. [18].

### Databases

For single nucleotide variation data we used the Exome Aggregation Consortium browser (beta version) ([11]; Exome aggregation consortium (ExAC), Cambridge, MA (URL: http://exac.broadinstitute.org/) accessed August 2016). The ExAC is a coalition of investigators seeking to aggregate and harmonize exome sequencing data from a wide variety of large-scale sequencing projects, and to make summary data available for the wider scientific community. The data set provided on the above mentioned website spans 60,706 unrelated individuals sequenced as part of various disease-specific and population genetic studies and 17 projects contribute to the data.

Short linear motifs were identified using the Eukaryotic Linear Motif (ELM) resource [19]. For information about catalogue of human mutations and related genetic disorders, the Online Mendelian Inheritance in Man^®^ was used (Online Mendelian Inheritance in Man, OMIM^®^. McKusick-Nathans Institute of Genetic Medicine, Johns Hopkins University (Baltimore, MD), {07/16/2016}. World Wide Web URLs: http://omim.org/). MIM Numbers and accession dates: F13a: {134570}{07/16/2016}; TGM1: {190195}{07/16/2016}; TGM5: {603805}{07/16/2016}; TGM6: {613900}{05/08/2016}. This article only uses anonymous human data from freely available public databases that have been obtained in a manner conforming to the respective IRB and/or granting agency ethical guidelines.

The mis_z scores were taken from the ExAC dataset [11]. In order to create a constraint metric and to contrast observed and expected number of variants per gene, mis_z scores were calculated. The mis_z score is based on a previous mutational model of probabilities of mutation for regional genomic divergence between humans and macaques [20] but with some modifications [11]. In Lek et al. [11] instead of probability of mutation, the expected number of variants were adjusted for depth of sequencing coverage for each exon. This depth adjusted correction was implemented to account for poorly sequenced regions with fewer variants than expectation. Across canonical transcripts all exon level variant counts were added and then a chi-squared value (Chi-square is a statistical test commonly used to compare observed data with data one would expect to obtain according to a specific hypothesis) for each mutational types (synonymous, missense, and protein-truncating) was calculated. If the observed variants are smaller than expected the square root of the chi-squared values were multiplied by 1 and if the observed variants are greater than expected then the values were multiplied by -1 to create a Z score. Finally, a corrected missense z score was created by dividing all missense z scores by standard deviation of the mirrored distribution. Mirrored or Gumbel distribution is an Extreme Value distribution Type-I; it is used for modelling extreme values of a random variable when the mean of smaller and larger values are farther apart.

Gene damage index (GDI) scores were taken from [21]. GDI is a metric which defines the non-synonymous mutational load in each protein-coding gene in the general population [21]. The damage caused to the exonic regions of the gene was predicted by calculating GDI score for each human gene g with n minor alleles (minor allele frequency < 0.5). For Phred I-score the ranking of the gene of interest i relative to all other human genes T = 19,558 genes, with values ranging from 0 (lowest Phred) to 42.91 (highest Phred) was calculated: I_i_ = −10[log 10(i/T)].

### Bioinformatic tools

To predict the possible impact of single amino acid substitutions on the structure and function of a human protein, Polymorphism Phenotyping (PolyPhen) and Sorting Intolerant From Tolerant (SIFT) analyses were applied for nsSNVs in the ExAC database. If more than one change is present at an amino acid residue and both having same scores, PANTHER (Protein ANalysis THrough Evolutionary Relationships) prediction was carried out to determine which of the two was the damaging nsSNV [22]. The deleterious, possibly or probably damaging nsSNVs are mentioned as damaging in the text.

GORIV was used to predict the secondary structures (https://npsa-prabi.ibcp.fr/cgi-bin/npsa_automat.pl?page=/NPSA/npsa_gor4.html accessed on 15 March 2016) of wild type and mutant human TGM2 proteins. Stability analysis was performed by algorithm FoldX [23] using default parameters of the program. Changes in the overall stability of the proteins (ΔΔG [kcal/mol]) for damaging nsSNVs were calculated in both opened (PDB ID: 2Q3Z) and closed (PDB ID: 1KV3) forms. TGM2 involving residues 1–14 and 323–326 for which the coordinates in either or both crystal structures are not available were excluded from the stability analysis.

### Homology modelling of TGM2

Homology models of TGM2 to represent one Ca^2+^ and a three Ca^2+^-bound states were built with SWISS-MODEL using the X-ray crystal structures of either FXIIIa (pdb: 4KTY) or TGM3 (pdb: 1L9M), respectively, as templates [24, 25, 26]. The models were then repaired for energy minimization using the FoldX forcefield. The R222 residue was mutated to glutamine using FoldX and the structures were visualized with the help of the FoldX plugin in YASARA [27, 28]. Figures were prepared using PyMol Molecular Graphics System (version 1.8 Schrödinger LLC).

### Transglutaminase enzyme preparations

The TGM2 variants were constructed using the QuikChange II Site-Directed Mutagenesis Kit Manual (Stratagene, La Jolla, California, USA) and were checked by restriction analysis and DNA sequencing (Capillary sequencing runs were performed by Genomic Medicine and Bioinformatics Core Facility at University of Debrecen). Wild-type TGM2 and homozygous nonsynonymous variants were expressed in N-terminally (His)_6_-tagged form (pET-30 Ek/LIC-TGM2) and purified by Ni-NTA affinity chromatography as described previously [29]. The protein concentrations were determined based on Bradford method (Bio-Rad Protein Assay, Bio-Rad, München, Germany). Finally, protein purity was checked after staining of SDS-polyacrylamide gels by PageBlue Protein Staining Solution (Thermo Fisher Scientific, Waltham, Massachusetts, USA). GenBank Accession Number of the wild-type TGM2 used in this study: RefSeq NM_004613. The dbSNP identifiers of the TGM2 homozygous nonsynonymous variants: p.Arg76His (rs41274720), p.Arg222Gln (rs200551434), p.Arg433Gln (rs142184177), p.Val542Phe (rs115436227), p.Pro612Thr (rs199563008), p.Asp671Asn (rs141236503).

### Transamidase assays

The kinetic amine incorporation assay was performed with slight modification of a previously published procedure [29]: The reaction mixture contained 50 mM Tris-HCl buffer pH 7.5, 0.5 mM dansyl-cadaverine, 2 mg/ml N,N’ dimethylated casein, 3 mM DTT, and 3 mM CaCl_2_ or 10 mM EDTA. The reaction volume was 100 μl and the assay was started with the addition of 20 μl enzyme (100 nM TGM2 final concentration). The reaction rate was calculated based on the initial slopes of the kinetic curves (Ex/Em: 360/490 nm; at 37°C) using GraphPad Prism version 7.00 for Windows (GraphPad Software, La Jolla, California USA, www.graphpad.com).

A real time fluorescence anisotropy assay was also used to measure the crosslinking activity [17]. The crosslinking of FLpepT26 into S100A4 (GST) by TGM2 was monitored by increase in the fluorescence anisotropy. The volume of the reaction mixture was 35 μl and performed for 30 mins at 37°C with 100 nM FLpepT26, 13.15 μM S100A4 (GST), 10 nM TG2, and 3 mM CaCl_2_ (5 mM EDTA was used as negative control). The reaction buffer contained 20 mM Tris–HCl, pH 7.5, 150 mM NaCl, 5 mM DTT, and 0.01% Tween 20. A 384-well untreated Polystyrene Black Microplate was used (Nunc, Thermo Scientific, Denmark, cat. no. 262260) and the change of the fluorescence anisotropy was measured by Synergy H1 microplate reader (GreenFP filter cube, excitation 485 nm, emission 528 nm; BioTek, Winooski, VT, USA). The reaction rate was calculated from the initial slopes of the kinetic curves using GraphPad Prism version 7.00 for Windows (GraphPad Software, La Jolla California, USA, www.graphpad.com).

### Isopeptidase assays

The basis of the Zedira assay is that transglutaminase cleaves the isopeptide bond in the synthesized substrate releasing the dark quencher (2, 4-dinitrophenyl) linked to the cadaverine spacer followed by the increase of fluorescence from the N-terminally attached fluorophore 2-aminobenzoyl (2-Abz). The reaction was performed based on previously published method [29]. In detail the reaction mixture was: 50 mM MOPS buffer, pH 6.8, containing 5 mM CaCl_2_, 100 mM NaCl, 0.1% (w/v) PEG8000, 100 nM TGM2, 50 μM A102 isopeptidase assay substrate (Zedira, Darmstadt, Germany), and 2.8 mM DTT (added with the starting solution which contained the enzyme). The reaction was monitored at 37°C by a Synergy H1 microplate reader (Ex/Em: 318/413 nm) and the activities were calculated from the initial slopes of the kinetic curves.

The isopeptidase assay using novel crosslinked protein–peptide substrate was carried out using previously published real-time fluorescence method [18]. The cleavage of the isopeptide bond on FLpepT26–S100A4 (GST) substrate by TGM2 was followed by decrease in fluorescence anisotropy. The volume of the reaction was 35 μl and performed for 60 mins at 37°C with 0.5 μM FLpepT26–S100A4 (GST), 300 nM TGM2, and 5 mM CaCl_2_ or EDTA. The reaction buffer contained 20 mM MOPS (pH 6.8), 150 mM NaCl, 6 mM glycine methyl ester, 5 mM DTT, and 0.1% Tween 20. A 384-well untreated Polystyrene Black Microplates (Nunc, Thermo Scientific, Denmark, cat. no. 262260) and Synergy H1 microplate reader (GreenFP filter cube, excitation 485 nm, emission 528 nm; BioTek, Winooski, VT, USA) was used to measure changes of fluorescence anisotropy. The reaction rates were calculated from the initial slopes of the kinetic curves in terms of anisotropy per minute using GraphPad Prism version 7.00 for Windows (GraphPad Software, La Jolla California USA, www.graphpad.com).

### BODIPY FL GTPγS nucleotide binding assay

500 nM BODIPY FL GTPγS, (Invitrogen, Carlsbad, CA, United States) [29] a GTP analog was used to compare nucleotide binding to increasing amounts of TGM2 variants in the presence of 20 mM HEPES pH 7.5, 150 mM NaCl, 0.1 mM TCEP, 0.05% Tween-20, 0.1 mM EGTA, and 1 mM MgCl_2_. There is an increase in fluorescence when BODIPY FL GTPγS binds to GTP-binding proteins providing a non-radioactive alternative tool to analyse protein-nucleotide interactions.

### Fibronectin binding assay

The fibronectin-binding property of the variants was tested using a previously published direct ELISA assay [16] with the following modifications. After fibronectin coating and washing, 0.3 μg TGM2 wild-type or variant was incubated in each well for 1 h at room temperature in TTBS buffer containing 2.5 mM CaCl_2_. The amount of bound TGM2 variants were detected using CUB7402 (1:5,000, Neo markers, Fremont, CA) monoclonal antibodies and then anti-mouse IgG/ HRP (1:7,500) with TMB substrate at 450 nm.

## Results

### Synonymous, non-synonymous and LOF nucleotide variants in genes of the human transglutaminase family

**Fig 1:**
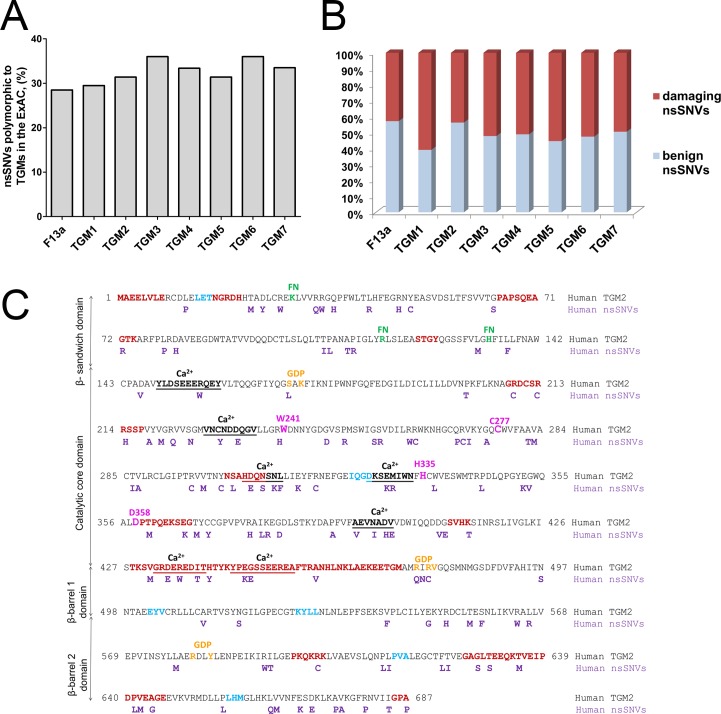
Analyses of non-synonymous variants in the transglutaminase family. **(A) Amino acid residues polymorphic to non-synonymous variants.** The percentage of polymorphic residues was obtained as a ratio of the total number of amino acid residues polymorphic to nsSNVs and the sequence length. The percentage of polymorphic residues is shown. **(B**) **Proportion of damaging and benign non-synonymous variants by the PolyPhen score.** Ratio of damaging nsSNVs: F13a (42.8%), TGM1 (60.8%), TGM2 (43.7%), TGM3 (52.2%), TGM4 (51%), TGM5 (55.3%), TGM6 (52.5%), and TGM7 (49.5%). **(C) Location of damaging nsSNVs in amino acid sequence of TGM2 by PolyPhen/SIFT scores.** Lane 1: sequence of human TGM2, Lane 2: damaging nsSNVs in human TGM2. Functional regions of human TGM2: Intrinsically disordered regions (dark red) [4], amino acid clusters in light blue [30], fibronectin binding sites (FN) (green) K30, R116, and H134 [31], GDP binding residues (orange) S171, K173, R476, R478, V479, R580, Y583 [32], catalytic residues (pink); non-canonical Ca^2+^-binding sites: S4: 149–159, S1: 228–236, S3A: 305–311, S3B: 326–333, S2A: 395–401, S5: 432–440, and S2B: 445–455 (underlined and bolded) [33]. Domains of human TGM2 are presented vertically: β sandwich (1–139), catalytic core (147–460), β-barrel 1 (472–583), and β-barrel 2 (584–687).

Recent advances in next-generation sequencing technologies and initiatives such as the 1000 Genomes and other exome sequencing projects have uncovered a broad range of genetic variations among individuals. For analysis of variants in the transglutaminase enzyme family the ExAC database was chosen as a source of SNV data. As of August 2016 it contained high quality exon sequencing data from 60,706 unrelated individuals displaying one variant at every eight bases [11]. There were 5,766 SNV entries for transglutaminases of which 3623 SNVs fall under synonymous, non-synonymous or LOF categories in exons (Table 1). Out of the total entries only 4.5–6% of SNVs in case of each family were non-synonymous SNVs. TGM2, TGM4, TGM5 had the lowest and TGM6 had the highest number of nsSNVs (Table 1). The range of loss-of-function (LOF; including frameshift, splice acceptor and stop gained) variants were from 19 in TGM1 to 40 in TGM5, while TGM2 had 29 LOF variants (Table 1). The number and percentage of residues polymorphic to nsSNVs in each family was also calculated. F13a, TGM1, TGM2, and TGM5 had less percent of such residues while TGM3 and TGM6 had the highest (Fig 1A).

**Table 1:** Number of different single nucleotide variants and gene constraints in human transglutaminase genes.

Gene	Synonymous	Non-synonymous	Loss-of-Function	n_mis	exp_mis	mis_z	GDI	GDI-Phred I
F13a	120	283	22	252	269.8	0.53	4254.7	12.9
TGM1	163	320	19	314	349.2	0.92	277.2	3.5
TGM2	148	272	29	270	290.5	0.59	240.6	3.3
TGM3	144	313	20	304	271.9	-0.95	1529.8	7.2
TGM4	121	279	39	264	232.6	-1.0	3544.7	11.4
TGM5	95	273	40	261	255.9	-0.15	2224.6	8.6
TGM6	136	336	39	320	287.7	-0.92	764.5	5.4
TGM7	91	293	28	283	263.3	-0.59	698.4	5.2

Number of synonymous, non-synonymous and LOF variants were determined based on data available in ExAC database. The n_mis, and exp_mis scores [11], and GDI and GDI-Phred I values [21] were taken from datasets published previously. See methods section for brief description and ref. [11] and [21] for detailed explanation of calculations and determination of the scores. Frameshift mutations, splice acceptor, splice donor, stop gained are included under LOF category. Definition of values and scores shown in the table is as follows. The **n_mis** scores: number of rare (minor allele frequency (MAF) <0.1%) missense variants found in ExAC r0.3 database. The **exp_mis** scores: depth adjusted number of expected rare (MAF <0.1%) missense variants. The **mis_z** scores: corrected missense z scores. The z score is a constraint metric to contrast observed and expected number of variants per gene [11]. The number of nsSNVs in columns 3 and 5 differ because for mis_z score calculation only nsSNVs with MAF < 0.1% are taken into account (n_mis, column 5). GDI is a metric used to score accumulated mutational damage on human genes [21]. GDI-Phred I: the GDI ranking of the gene relative to all other human genes.

### TGM1, TGM2 and F13a show evolutionary constraint to non-synonymous variants

In order to determine the constraint on a particular gene, mis_z scores were calculated for non-synonymous variations of each gene in the ExAC dataset [11]. The mis_z values for the transglutaminase nsSNVs are given in Table 1. Increased constraint is indicated by positive mis_z scores and decreased constraint with negative mis_z scores. TGM1, TGM2 and F13a had positive, highest mis_z scores and, therefore, they had fewer variants than expected but other members had negative mis_z scores indicating that they had more variants than expected (Table 1).

Recently, by using gene level approach, load of disease causing mutations and mutational damage on protein-coding human genes was estimated by gene damage index scores (GDI) [21]. The genes with low GDI score tend to be under strong purifying selection and biologically indispensable and thus harbor fewer mutations. But those with high GDI scores are under less purifying selective pressure and are likely biologically redundant with more mutations. Based on Phred I-score, the ranking of the gene of interest i relative to all other human genes (T = 19,558 genes used in the analyses in ref [21]) was also calculated to estimate the damaged human genes. Lowest Phred I-score refers to least damaged human gene with lowest GDI and highest Phred I-score refers to most damaged human gene. The GDI and GDI-Phred I values are listed for transglutaminases in Table 1. Among transglutaminases, TGM2 and TGM1 with lowest values are under strong selective pressure as they cannot tolerate more mutations. Mutations are tolerated by other transglutaminases, particularly TGM4 and F13a with highest Phred I-score. F13a is under less purifying selection based on the GDI values, but F13a deficiency results in bleeding disorder [34].

### TGM2 and F13a have lowest ratio of damaging non-synonymous SNVs

The SIFT and polymorphism phenotyping scores provided in the ExAC database were used. According to SIFT analysis, F13a and TGM6 have lowest and highest ratios of damaging nsSNVs, respectively (data not shown). PolyPhen analysis revealed that TGM2 and F13a have the lowest, while TGM1 and TGM5 have the highest ratios of damaging nsSNVs (Fig 1B). Around 45% of nsSNVs in TGM2 are damaging and out of these 54% are concentrated in the catalytic core domain and 17% in the β-sandwich domain (Fig 1C).

### Effect of damaging nsSNVs on TGM2 structure and function

The damaging nsSNVs affect the stability and biochemical functions of the native proteins [35]. We analyzed how rare PolyPhen or SIFT predicted damaging nsSNVs (indicated in Fig 1C) influence protein stability, secondary structure and functional sites including novel amino acid clusters, IDRs, SLiMs, and LC3 (microtubule-associated protein light chain) interacting regions (LIRs).

#### Influence on stability and secondary structures

Stability effects of mutations are crucial for understanding the sequence–structure relationships and predicting the evolutionary dynamics of proteins [36]. FoldX analysis to assess the impact of damaging nsSNVs on TGM2 stability was performed using both the opened (PDB ID: 2Q3Z) and closed (PDB ID: 1KV3) conformations of TGM2. Mutations had equal impact on relative stability (ΔΔG) of the opened and closed conformation. Accordingly, 24.4% of nsSNVs in the opened conformation and 28.6% in the closed conformation were found to be destabilizing (ΔΔG > 1 kcal/mol), 26.7% and 23.5% were highly destabilizing (ΔΔG > 3 kcal/mol) in opened and closed conformation, respectively. The highly destabilizing damaging nsSNVs are dominantly located in the catalytic core domain, in accord with the observation that mutations affecting the function of a protein are highly destabilizing [37].

Based on GORIV predictions only eight damaging nsSNVs had a minor impact on the secondary structure propensities: only the insertion of a helix-breaking proline at position 12 induced helix to coil transition (S1 Table). The persistence of secondary structures after generation of nsSNVs also underscores the stability of the human TGM2 structure. Variants, p.Arg214His and p.Trp337Leu had destabilizing ΔΔG values in the opened and closed conformations and p.Arg377His in the closed conformation (S1 Table). The ΔΔG value of p.Val283Met is highly destabilizing in the opened conformation.

#### Effect on conserved functional and interaction sites

Damaging nsSNVs are not present at active site residues (W241, C277, H335, and D358) and at recently described novel amino acid clusters emerged in humans [30]. We have previously identified additional residues around the active site, which are crucial for transamidase (W278) or isopeptidase (W332) activity [29] and there are no damaging nsSNV at these sites either. Localization and function of TGM2 in the extracellular space is critically determined by its interaction with fibronectin; there is no nsSNVs at the recently described fibronectin binding residues in TGM2 [31].

Damaging nsSNVs p.Ser171Leu, p.Arg476Gln, and p.Arg478Cys slightly influence the GDP binding sites [32] and all of the described non-canonical calcium binding sites [33] are affected (Fig 1C). Studies indicate that non-enzymatic protein-protein interactions of TGM2 have important physiological and pathological outcomes (reviewed in [4]). The described interaction sites in TGM2 were checked for the presence of damaging nsSNVs. Syndecan-4, which functions as a receptor for intracellular signaling is reported to interact with sequence ^202^KFLKNAGRDCSRRSSPVYVGR^222^ of TGM2 [38, 39] which has six nsSNVs. Likewise, interaction sites of various proteins in TGM2 enclose damaging nsSNVs: the α1-adrenoceptor interaction sites L547-I561, R564-D581, and Q633-E646 [40, 41] embed eleven nsSNVs, the PLCδl interaction sequence V665-K672 [42, 43] has three nsSNVs, BAX and BAK interaction sequence 204–212 [44] embeds one, 14-3-3 binding protein interaction sequence 209–223 [45] has seven nsSNVs and three SUMO motifs detected on TGM2 sequence 327–329, 364–366, and 468–470 [46] has two nsSNVs. Therefore, presence of nsSNVs in regions targeted by several interacting partners might influence cellular TGM2 functions in transmembrane signaling, cell adhesion, migration, Ca^2+^ regulation of transamidation, cell death induction, and protein turnover.

The damaging p.Met330Arg nsSNV is among the three reported heterozygous missense mutations in the TGM2 gene associated with early-onset type 2 diabetes in a small disease cohort [47, 48]. It should be noted that association of TGM2 mutations and dysfunction has not been confirmed in larger diabetes patient cohorts so far and TGM2 KO mice have no impairment in glucose-stimulated insulin secretion by pancreatic islets relative to wild-type littermates [6].

TGM2 has a pivotal role in celiac disease pathogenesis through generating immunogenic peptides from gliadin and because of the appearance of pathologic anti-TGM2 antibodies during the course of the disease. It is interesting to see that there is practically no damaging nsSNV located in the identified celiac epitope relevant amino acid residues, namely epitope 1 which is composed of Lys30, Arg116, His134 and epitope 2 consisting of Arg19, Glu153, Glu154, Met659 [49, 50].

#### Occurrence of nsSNVs at intrinsically disordered regions embedding short linear motifs

The sequences of many proteins contain short, loosely-defined protein interaction sites that mediate recognition and targeting activities and provide wide range of functionality to proteins [51]. These interaction sites are called Short Linear Motifs (SLiMs) composed of low complexity short peptide regions (3–20 residues long) which mediate post-translational modifications and protein-protein interactions. Mostly SLiMs are embedded in polypeptide segments that lack well-defined tertiary structure. These unstructured regions are referred to as intrinsically disordered regions (IDRs) and they are involved in diverse functions. About 22% of human disease mutations occur in intrinsically disordered regions [51]. Recently we reported 13 IDRs embedding 39 SLiMs in human TGM2 [4] and we looked for the presence of damaging nsSNVs in these regions. The damaging nsSNVs located in IDRs embedding SLiMs are given in Table 2. They target 8 IDRs embedding 27 SLiMs and most of the nsSNVs are concentrated in the IDRs 208–217, 411–414, and 428–473 located in the catalytic core domain (Table 2).

**Table 2:** Damaging nsSNVs located in ID regions embedding SLiMs in TGM2.

IDR	Short linear motifs	Explanation	Damaging nsSNVs
65–74	• LIG_SH3_3 [59–65]	SH3 binding domain	• p.Gly64Ser
208–217	• DOC_MAPK_1 [213–220] • DOC_WW_Pin1_4 [213–218] • MOD_GSK3_1 [209–216] • MOD_CK1_1 [212–218] • MOD_PKA_2 [212–218] • MOD_ProDKin_1 [213–219]	Docking interaction in MAP kinase cascade (exemplified cJun); binds WW domains, involved in proline directed phosphorylation signaling pathways; phosphorylation motifs (CK1, GSK3,PKA, proline-directed kinase)	• p.Arg209Cys • p.Arg213Cys • p.Arg214His • p.Val218Ala • p.Val220Met
358–367	• DOC_WW_Pin1_4 [357–362] • LIG_FHA_2 [358–364] • LIG_TRAF2_1 [360–363] • LIG_TRAF6 [359–367] • MOD_CK2_1 [357–363] • MOD_ProDKin_1 [357–363]	Binds WW domains, involved in proline directed phosphorylation signaling pathways; TRAF2 and TRAF6 binding motif; CK2 and proline-directed kinase phosphorylation motif	• p.Thr360Met • p.Glu366Lys
411–414	• LIG_ACTIN_WH2_2 [409–427] • MOD_GSK3_1 [408–415] • MOD_PLK [408–415]	Actin binding motif (WH2 domains); GSK3 and PLK phosphorylation site;	• p.Asp409Val • p.Asp409Gly • p.Gly410Glu • p.Lys414Thr
428–473	• DOC_USP7_1 [446–450] • MOD_CK1_1 [427–433] • MOD_CK2_1 [446–452]	USP7 binding motif; CK1 and CK2 phosphorylation motif	• p.Glu447Lys • p.Gly448Glu • p.Val431Leu • p.Val431Met
597–602	• TRG_LysEnd_APsAcLL_1 [599–604] • TRG_NLS_MonoExtN_4 [597–604] • DOC_CYCLIN_1 [601–604] • DOC_MAPK_1 [601–609]	Sorting and directing signal to lysosomal endosomal compartment; NLS; cyclin recognition signal; MAPK docking motif	• p.Arg601Cys
626–647	• DEG_APCC_DBOX_1 [650–658] • DOC_MAPK_1 [649–657] • LIG_FHA_1 [633–639]	APCC binding destruction signal; MAPK docking motif; FHA binding motif	• p.Pro656Leu • p.Thr635Met
685–687	• DOC_MAPK_1 [674–684]	MAPK docking motif	• p.Ala675Pro • p.Val676Ala • p.Arg680Pro • p.Ile684Thr

Column 1 indicates the position of IDRs; column 2 shows the names of SLiMs and their coordinates in square brackets; column 3 provides explanation for the function of the given SLiM; column 4 lists damaging nsSNVs in TGM2.

#### Occurrence of nsSNVs at LC3 interacting regions

Autophagy was initially considered to be a nonselective process for bulk breakdown of cytosolic material. However, recent evidence points towards a selective mode of autophagy mediated by the so-called selective autophagy receptors [52]. Interaction between selective autophagy receptors and proteins of the autophagy-related protein 8 families are mediated by short linear sequence motifs called LIRs which ensures the targeting of autophagy receptors to LC3 or other autophagy-related protein 8 family proteins anchored in the phagophore membrane. The canonical LIR motif consists of a short tetrapeptide sequence WxxL (where x could be any residue), which interact with two distinct hydrophobic pockets of LC3 [52]. As TGM2 was shown to be involved in autophagy [53], SNVs in LIRs motifs could impact this process. LIR motifs and 11 damaging nsSNVs in TGM2 are shown in Table 3. Two damaging nsSNVs target xLIR motif ^352^EGWQAL^357^ and WxxL motifs are targeted by nine damaging nsSNVs (Table 3). Non-synonymous SNVs present within LIRs might have functional implications in autophagic process, given the role of TGM2 in autophagosome maturation [53] and its interaction with autophagy cargo proteins [54].

**Table 3:** Damaging nsSNVs located in potential LC3 interacting regions.

MOTIF	START	END	LIR sequence	TGM2 damaging nsSNV
xLIR	352	357	**EG**WQAL	• p.Glu352Lys • p.Gly353Val
WxxL	38	43	PFWLT**L**	• p.Leu43Arg
WxxL	133	138	GHFI**L**L	• p.Leu137Phe
WxxL	278	283	WVFA**AV**	• p.Ala282Thr • p.Val283Met
WxxL	392	397	FVF**A**EV	• p.Ala395Val
WxxL	514	519	VSY**N**GI	• p.Asn517Ser
WxxL	677	682	KGF**R**NV	• p.Arg680Trp • p.Arg680Pro • p.Arg680Gln

Column 1 represent the LIR and WxxL motifs, column 2 and 3 represent the start and end of each motif in TGM2, column 4 represent the sequence of LIR motifs and human TGM2 damaging nsSNVs are underlined in bold text.

### Population scale occurrence of non-synonymous SNV in TGMs

**Fig 2:**
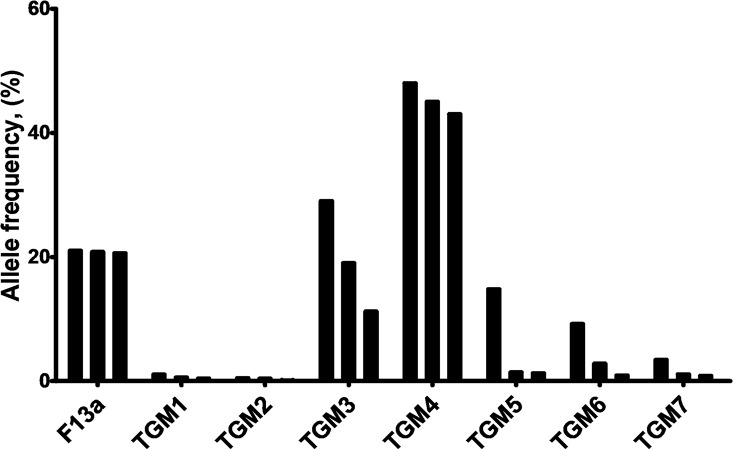
Allele frequencies of three most frequent non-synonymous variants in transglutaminase family genes. Values were calculated based on the ExAC database. For each family, nsSNVs with top three allele frequency (%) values are shown. **F13a**: p.Pro565Leu (21%), p.Glu652Gln (20.8%), p.Val35Leu (20.6%); **TGM1**: p.Val518Met (1.04%), p.Glu520Gly (0.56%), p.Ser42Tyr (0.40%); **TGM2**: see in text; **TGM3**: p.Gly654Arg (28.7%), p.Thr13Lys (19%), p.Ser249Asn (11.2%); **TGM4**: p.Glu313Lys (49%), p.Val409Ile (44.7%), p.Arg372Cys (42.8%); **TGM5**: p.Ala352Gly (14.8%), p.Val504Met (1.4%), p.Gln521Arg (1.25%); **TGM6**: p.Met58Val (9.2%), p.Arg448Trp (2.80%), p.Ala141Glu (0.9%); **TGM7**: p.Pro564Leu (3.4%), p.Val103Leu (1.07%), p.Val515Leu (0.82%).

The influence of nsSNVs of proteins arising in the human population is significantly determined by their penetrance. The nsSNV allele counts in the population covered in the ExAC dataset are the highest for TGM3 and TGM4 (over 0.2 million) and lowest for TGM2 and TGM1 (2601 and 5174, respectively) (Table 4). Common variants are typically defined as those found with > 5% allele frequency and rare variants as those found with < 0.5% allele frequency [55]. The F13a, TGM3, TGM4, TGM5, and TGM6 have common nsSNV variants, TGM1 and TGM7 nsSNV variants occur in frequencies less than 5%. The allele frequency value for the three most frequent variants in case of each family member is showed in Fig 2. All the TGM2 nsSNVs were rare with allele frequency values less than 0.5%. The TGM2 nsSNV with highest allele frequency values are p.Arg76His (0.47%), p.Val542Phe (0.38%), p.Glu366Lys (0.11%), p.Arg433Gln (0.10%) and p.Glu469Gly (0.10%); of these only p.Val542Phe has potentially damaging PolyPhen score.

**Table 4:** Summary of population frequencies of nsSNVs alleles of transglutaminases in the ExAC database covering 60,706 individuals.

Genes	Total number of nsSNV types	Allele count	Number associated with homozygotes	Number of homozygote individuals
F13a	283	88654	9	9214
TGM1	320	5174	13	25
TGM2	272	2601	6	12
TGM3	313	205153	12	73495
TGM4	279	265931	17	66931
TGM5	273	27038	12	2206
TGM6	336	111028	23	47462
TGM7	293	11585	13	170

Column 2 shows the total number of nsSNVs; column 3 has the observed allele counts for the nsSNVs; column 4 presents the number of nsSNVs associated with homozygotes; column 5 contains the number of homozygote individuals

### Homozygous occurrence of TGM2 non-synonymous SNVs

Since damaging TGM2 nsSNVs occur at some functional sites of the protein we have decided to screen databases to see whether homozygous occurrence of nsSNVs is tolerated by individuals as compared to nsSNV variants of the other transglutaminase family members. Furthermore, we wanted to learn how the protein products of TGM2 nsSNVs alleles found in homozygotes function in biochemical tests.

The current ExAC dataset contains the revealed existing homozygous nsSNVs for transglutaminase family members (Table 4). TGM3, TGM4, and TGM6 have the highest (in the range of 47 to 73 thousands), while TGM2, TGM1, and TGM7 have the lowest (12, 25, and 170 respectively) numbers of such homozygous occurrences (Table 4). The number of nsSNV alleles associated with the homozygotes is lowest for TGM2 and F13a. There are only six TGM2 nsSNVs which occur all together in 12 individuals in homozygous form in various populations in the World, by far the lowest number in the TGM family (Table 5). Amidst these, three nsSNVs have probably damaging PolyPhen scores (p.Arg222Gln, p.Val542Phe and p.Pro612Thr), p.Pro612Thr is destabilizing in both closed and opened conformations, whereas p.Val542Phe and p.Asp671Asn mostly affect the closed conformation (Table 5).

**Table 5:** The 12 human TGM2 homozygotes related to 6 nsSNVs.

Position and Change	Allele Frequency [%]	Domains	Stability ΔΔG [kcal/mol]		PolyPhen/ SIFT	Number of Homozygote Individuals	Population
			Closed Form	Open Form			
R76H	0.47	β-sandwich	0	0.57	Benign	5	• 2 East Asian • 3 Latino
R222Q	0.048	Catalytic	-0.98	0.71	Probably damaging	1	• South Asian
R433Q	0.10	Catalytic	0.67	0.51	Benign	1	• South Asian
V542F	0.38	β-barrel 1	2.29	-1.11	Probably damaging	3	• 2 East Asian • 1 African
P612T	0.018	β-barrel 2	2.26	3.83	Probably damaging	1	• South Asian
D671N	0.013	β-barrel 2	1.17	0.10	Benign	1	• African

Column 1 homozygous nsSNVs in TGM2; column 2 allele frequency of the given nsSNV; column 3 shows domain location in TGM2; column 4, 5 stability values in closed and opened conformation; column 6 PolyPhen/SIFT scores; column 7 presents number of homozygote individuals for each nsSNV and column 8 indicates population distribution of the 12 homozygote individuals. The dbSNP identifiers of the TGM2 homozygous nonsynonymous variants: p.Arg76His (rs41274720), p.Arg222Gln (rs200551434), p.Arg433Gln (rs142184177), p.Val542Phe (rs115436227), p.Pro612Thr (rs199563008), p.Asp671Asn (rs141236503).

### Biochemical analysis of homozygous non synonymous TGM2 variants

**Fig 3:**
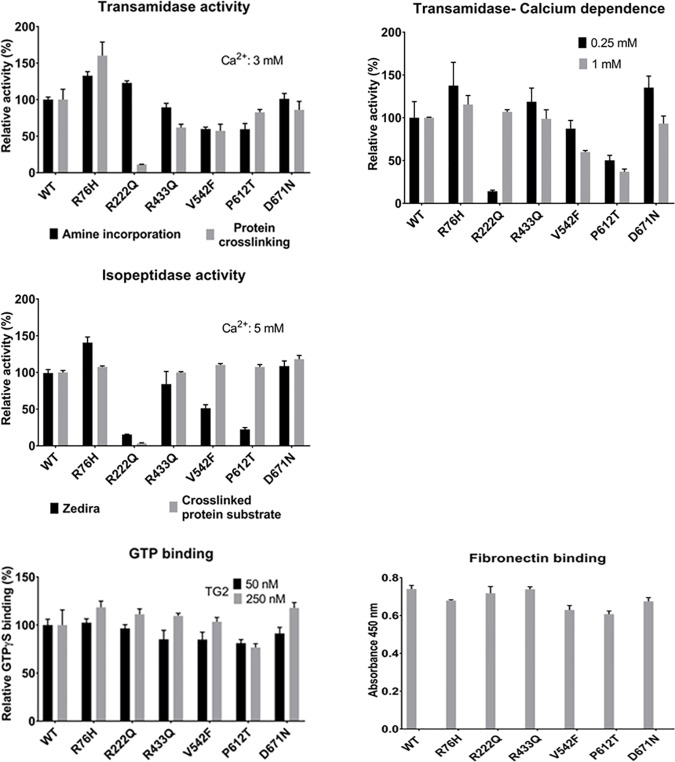
Biochemical characterization of the six homozygous nsSNVs of TGM2. Kinetic characterization of the transamidase activity of TGM2 variants and wild type at 3 mM CaCl_2_ concentration using amine incorporation assay [56] and protein crosslinking assay [17]. Calcium dependence of kinetic transamidase reaction with 0.25 and 1 mM calcium concentrations using amine incorporation assay. Comparison of the kinetic isopeptidase activity of TGM2 variants at 5 mM CaCl_2_ concentration using Zedira assay and crosslinked protein substrate [18]. The relative activities are calculated as a percentage of the activity values of the wild type TGM2. Comparison of BODIPY FL GTPγS binding of variants and wild type TGM2 proteins with different concentrations of TGM2 (50 and 250 nM). The change in the fluorescence intensity (Ex/Em: 485/520 nm) was determined after 15 minutes of incubation. Binding is shown as a percentage of maximum binding in case of wild type TGM2 [29]. The Fibronectin binding property of the TGM2 variants was tested using a previously published direct ELISA assay [16]. Data are presented as means with ± standard deviations from three separate experiments done in triplicate. All the data were analyzed by GraphPad Prism 7.

Since homozygous nsSNVs can be associated with various diseases we analyzed the functional impact of TGM2 homozygous nsSNVs. We produced all the six homozygous occurring TGM2 variants containing the respective nsSNVs by site directed mutagenesis and tested them in biochemical assays. We measured transglutaminase activity of the variants using two previously published kinetic assays [17, 56]. Except p.Arg222Gln, the transamidase activity of variants is comparable in the two assays. p.Arg222Gln was completely active in amine incorporation assay but inactive in the protein crosslinking assay (Fig 3). The variant p.Arg76His showed increase in transamidase activity compared to the wild type enzyme and variant p.Val542Phe showed 40% less activity than wild type TGM2 in both assays. Compared to the wild type, p.Pro612Thr variant showed 40% less activity in the amine incorporation assay but only 18% less activity in the protein crosslinking assay. Interestingly both p.Val542Phe and p.Pro612Thr variants have predicted damaging PolyPhen/SIFT scores (Table 5). Calcium dependence of the transamidase reaction was also measured based on the amine incorporation assay (Fig 3). Variant p.Arg76His exhibited high transamidase activity and variants p.Val542Phe, p.Pro612Thr had less activity than wild type at both measured Ca^2+^ concentrations (Fig 3). Variants p.Arg433Gln, p.Asp671Asn manifested a several fold increase in transamidase activity compared with wild type at 0.25 mM Ca^2+^ concentration. Variant p.Arg222Gln showed the lowest efficiency at 0.25 mM calcium concentration, increasing of which restored activity. It is known that Ca^2+^ and nucleotides reciprocally regulate transglutaminase reactions. The nucleotide binding of the variants were examined using an analog, BODIPY FL GTPγS. At 250 nM enzyme concentration, 18% higher GTP binding was observed for p.Arg76His and p.Asp671Asn than wild type and 24% lower GTP binding in case of p.Pro612Thr (Fig 3).

TGM2 also possess isopeptidase activity when previously formed isopeptide bonds are cleaved. The isopeptidase activity of the six variants was also compared using a commercially available small chemically produced substrate and a recently published protein based real-time kinetic method [18]. The p.Arg222Gln variant is only 15% active in the commercial Zedira assay and completely inactive in the protein based method (Fig 3). Compared to wild type, the variants p.Val542Phe and p.Pro612Thr showed less isopeptidase activity in the Zedira assay but normal activity in the protein based method. While variants p.Arg76His, p.Asp671Asn showed increase in activity compared to the wild type in both the assays. Since interaction of TGM2 with fibronectin has a crucial role in scaffolding processes in the extracellular matrix of the cells, the fibronectin-binding property of the variants was tested by an ELISA method. The variants p.Val542Phe, p.Pro612Thr showed 15% less fibronectin binding but other variants bound fibronectin similarly to the wild type enzyme (Fig 3).

Homozygous variants p.Arg76His and p.Asp671Asn with high transamidase, isopeptidase activities and GTP binding ability do not coincide with any of the functional sites, predicted IDRs, novel clusters and have benign or tolerated scores. The p.Arg433Gln variant with almost normal activity compared to wild type is part of the Ca^2+^ binding site S5 (432–440) and IDR (428–473) embedding SLiMs like USP7 binding motif, TRAF2 binding motif, and CK1 and CK2 phosphorylation motif. A PolyPhen damaging variant with decreased activity is p.Val542Phe located within the MOD CK1 phosphorylation site and MOD PLK site phosphorylated by polo like kinases. Another potentially damaging variant p.Pro612Thr with less transamidase, isopeptidase and GTP binding is near to an IDR (597–602) and a novel amino acid cluster PVA (613–615) [30]. The homozygous variant p.Asp671Asn is located in the C-terminal class 3 PDZ-binding motif.

**Fig 4:**
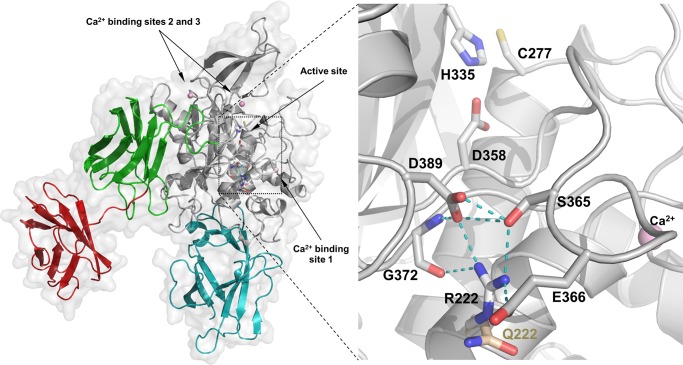
Structural interpretation of the effect of the p.Arg222Gln variation on enzyme activity. We analyzed the interactions of R222 in a homology model of TGM2 containing three bound calcium ions (pink spheres), which supposedly corresponds to the active form (cyan: N-terminal, grey: catalytic, green and red: first and second beta barrel domains respectively). The squared area in the left panel is magnified on the right. R222 is located in the middle of the solvent accessible surface of the α-helix leading up to calcium binding site S1 (226–233). R222 is at the core of an H-bond network (light blue dashed lines) that serves to bundle neighboring structural elements of TGM2 together. Upon binding of calcium to site 1, H-bonds between E232, N229 and backbone atoms of Y369, and H-bonds of R222 to S365, E366, G372, and D389 cooperatively tether the flexible loop, P359-G372. The changing conformation of this loop leads to reorganization of another non-covalent interaction network near calcium binding site 2, including a directly calcium binding residue, N306, for metal binding, and to honing of the charge relay duad, E305-E363 that has recently identified importance for catalysis [26]. The Q222 variant (wheat) fails to establish the critical H-bonds with S365, E366, and E389, thus the calcium binding of both sites are impaired and the charge relay system is also negatively affected. The same loop, most probably, also contributes residues to the amine substrate binding surface and controls access to the active site (C277/H335/D358), likely explaining that the Q222 enzyme has conserved transamidase activity for a small molecular amine, but compromised cross-linking activity towards a protein amine donor and lost isopeptidase activity for a protein-peptide conjugate.

The PolyPhen damaging variant p.Arg222Gln, located to the catalytic core domain near Ca^2+^ binding site S1 (226–233) and the SLiM, STAT5 Src Homology 2 (SH2) domain binding motif, has very low transamidase activity at physiological Ca^2+^ concentration. Moreover, the isopeptidase activity of this variant is completely lost. To assess the effect of the p.Arg222Gln allele on the structure we compared the situations where of the calcium binding sites none, only site 1, or all three are occupied by metal using the existing crystal structures and newly built homology models of TGM2 (Fig 4). We attribute the observed behavior of the Q222 variant to a disrupted H-bond network that leads to altered conformation of the loop, P359-G372, and consequently to reduced calcium affinities at site 1 and 2, and to a topology which disfavors proper interaction with a protein amine donor. Taken together, biochemical data demonstrates that only one of the very rarely occurring homozygous nsSNVs containing variant of TGM2 lead to significant decrease of one of its basic functional properties.

### Heterozygous and homozygous LOF variants of TGMs

The very rare generation of damaging nsSNVs alleles of TGM2 in humans and the observation that even the so far found homozygous TGM2 nsSNVs keep their basic biochemical properties (with one exception with decreased activities), prompted us to check the population frequency of its LOF variants compared to the other genes. Disease causing TGM2 LOF variants have not been reported so far in humans. Neither TGM2 nor the other TGMs are among the 3230 genes of the ExAC database which are unable to function normally after loss of one copy of the gene, even if the other copy is intact (haploinsufficiency) [11]. In general, large-scale sequencing projects revealed a surprisingly large number of LOF variants and fully knocked out (homozygous or compound heterozygous) genes in genomes of healthy individuals [57]. We compiled the LOF variants of transglutaminase family members from different populations with available data (S2 Table). Heterozygous LOF variants for all the transglutaminases were identified in the dataset of the Atherosclerosis Risk in Communities (ARIC) cohort study (S2 Table) [58]. In the ExAC dataset (where full knock outs can be found), LOF heterozygotes were identified for all the members of the family but in homozygous form only for TGM4 and TGM6 [11]. Transglutaminase LOF variants with highest allele frequency values were observed for TGM4 (0.83%) and F13a (0.24%) while the LOF (MAF) values were very low for the others: TGM1 (0.03%), TGM2 (0.015%), TGM3 (0.003%), TGM5 (0.006%), TGM6 (0.04%), TGM7 (0.017%). Exon sequencing data from 185 individuals as part of the pilot phase of the 1000 Genomes Project was analyzed and 2951 LOF variants, rare and likely deleterious LOF alleles (including 26 known and 21 predicted severe disease-causing variants) were reported [57]; this dataset contains a homozygous TGM6 LOF variant. A list of autosomal genes that are completely knocked out in the Icelandic bottlenecked population by rare LOF mutations have been published [10]; these data show that 7.7% (1171) of protein coding genes are completely knocked out by loss-of-function variants including TGM1, TGM4, and TGM5 (S2 Table). Recently, 3222 exomes of British adults of Pakistani heritage, a consanguineous population, was sequenced and 1111 rare gene knockouts in 781 genes were identified finding one homozygous TGM4 LOF variant [12]. A homozygous TGM4 LOF variant was also identified in the Simons Simplex Collection (SSC) dataset, a project aimed to improve the understanding and research of autism spectrum disorders [59]. TGM2, TGM3, and TGM7 LOF variants in homozygous or compound heterozygous form have not been identified in any of these datasets.

### Summary of transglutaminase mutations listed in OMIM database

Allelic variations responsible for human inherited diseases are listed in the Online Mendelian Inheritance in Man (OMIM) database, an Online Catalog of human genes and genetic disorders. We collected OMIM data for transglutaminase family which is shown in S3 Table. F13a autosomal recessive mutations lead to F13a deficiency [60, 61, 62], TGM1 has disease causing mutations in autosomal recessive congenital ichthyosis [63, 64], TGM5 homozygous LOF mutations cause acral peeling skin syndrome [65, 66, 67] and TGM6 mutations are associated with spinocerebellar ataxia 35 [68]. Data have not been found for inherited disease related to TGM2, TGM3, TGM4, and TGM7 mutations.

## Discussion

Large scale exome sequencing has opened a new avenue to study molecular background of polymorphism and to understand unsolved Mendelian and non-Mendelian genetic disorders [7, 69, 70, 71]. Our survey reveals that human transglutaminase genes harbor about the same number of nsSNV types in accord with the assumed generational mutation rate of ~1–1.5 × 10^−8^ for single-base substitutions, but differ very much in their response to purifying selection. Deleterious variants are expected to have lower allele frequencies than neutral ones, due to negative selection. Genomic changes in F13a, TGM3, TGM4, and TGM6 are most tolerated with highest nsSNV allele frequency values, allele number and homozygote individuals but are least tolerated in TGM1, TGM7 and particularly TGM2 with low population frequencies. Evolutionary constraints are also indicated by mis_z and GDI scores for TGM1 and TGM2 in the human population. These differences are even more intriguing knowing that TGM6, TGM3 and TGM2 are located close to one another on chromosome 20q11-12. We presume that based on population frequencies and other predicted values, human TGM2 must be under high selective pressure not allowing generation of even heterozygous common variants. The similarly high evolutionary constraint on human TGM7 is a surprising finding and may initiate further studies, especially to learn the physiological function of TGM7 about which there is no available information.

Rare, deleterious non-synonymous variants can be lethal and are also connected to human diseases [72]. Caution should be taken while addressing damaging variants as some previously identified disease-causing variants were actually benign occurring at high frequencies [11]. Recent studies have disclosed rare, homozygous LOF variants or knockouts in genomes of healthy individuals [57]. So far, 1717 genes have been reported to be implicated in various Mendelian recessive disorders (the most common is cystic fibrosis) [73–76]; the lack of most of these genes is compatible with life albeit with the consequence of disease phenotypes. Based on available datasets, F13a, TGM1-TGM7 have LOF variants in heterozygote state but homozygous LOF variants have been seen only for TGM1, TGM4, TGM5, and TGM6. From classical clinical and genetic studies it has been known that humans with knock out, homozygous LOF transglutaminase genes with disease phenotype in the case of F13a (bleeding disorder, prevalence 1 in 2 millions) [60, 61, 62, 77, 78], TGM1 (lamellar ichthyosis, prevalence 1 in 150 thousands) [63, 64], and TGM5 (acral peeling skin, prevalence <1 in 1 million) [65, 66, 67] deficiencies. It is important to note that TGM2, TGM3, and TGM7 nsSNVs do not seem to be associated with any pathological phenotype except perhaps TGM2 heterozygous mutations associated with early-onset type 2 diabetes, an observation which has not been confirmed in animal experiments and large clinical cohorts [6, 47, 48, 79]. The most plausible explanation of this remarkable purifying selection, at least for TGM2, is its multifunctional nature. TGM2 is able to catalyze transamidation, esterification, GTPase, protein disulphide isomerase, and hydrolysis reactions [1, 2], and interacts with a large number of ligands and proteins in almost all compartments and outside of cells (reviewed in [4, 5]). It is likely that almost all amino acids in the sequence have structural and functional importance and loss of any of them manifested in homozygotes would lead to deleterious consequences–even preventing full embryonic development. This conclusion and the fact that TGM2 knock-out mice are viable and are fertile raise the possibility that when compared to rodents, human TGM2 (and possibly also TGM3, TGM7) gained vital functions which have not been fully revealed, yet.

Information from literature unveil that disease causing SNVs are concentrated at the core of a protein [80]. Our study also shows that most of the TGM2 damaging nsSNVs are concentrated to the catalytic core domain. The core of the protein is hydrophobic in nature, so mutation of a hydrophobic residue to a charged or polar residue could destabilize the protein. Our stability analysis revealed that most of the highly destabilizing damaging nsSNVs are in the catalytic core domain. TGM2 nsSNVs in homozygous state so far found in humans and tested by us resulted in minor changes in biochemical activities but there were no deleterious consequences in basic structural and functional features. Only the p.Arg222Gln variant had reduced transamidase and isopeptidase activities in assays where the amine donor was a protein (S100A4). This variant also showed diminished responsiveness in the amine incorporation assay to a low but physiologically relevant range of Ca^2+^ concentration. We attribute this to the importance of the non-covalent interaction network around R222 in positioning the loop, P359-G372. This loop carries residues, which are engaged in calcium binding at site 1 as well as substrate interactions. Without stabilizing interactions with site 1 and 2 and R222 most of this loop is intrinsically disordered and highly dynamic. The disrupted H-bond network in the Q222 variant leads to altered conformation of the loop and in consequence, probably, to reduced Ca^2+^ affinities at sites 1 and 2, and to a topology which disfavors proper interaction with the protein amine donor.

None of the homozygous nsSNVs are located in conserved or unique novel functionally important sites of TGM2 which are not affected by other damaging nsSNVs either. The first cloned human recombinant TGM2 has glycine at position 224 [81] opposed to valine in the endogenous sequence present in the available genomic databases. We reported that the V224 variant of the enzyme has higher calcium-binding affinity, transamidation activity and isopeptidase activity [82] and it is important to note that there has been no nsSNV detected in the human population so far at this conserved Val224 residue either.

IDRs and SLiMs play a vital role in protein function, they provide conformational flexibility and facilitate diverse post-translational modifications and protein-protein interactions [83]. Recent studies have shown that disease-related mutations [84] and nsSNVs [85] are enriched in SLiMs in IDRs and they occur more frequently at functionally important residues of SLiMs [85]. Our study could reveal TGM2 damaging nsSNVs within IDRs embedding SLiMs (Table 2) suggesting that sequence variability at these sites may have functional significance, although almost all of these nsSNVs occur as heterozygous alleles. A damaging nsSNV in the β-sandwich domain affects the IDR 65–74 and a SLiM motif recognized by SH3 domains. In the catalytic core, series of six nsSNVs are located in IDR 208–217 and the SLiM motifs involved in proline directed phosphorylation signaling pathways. Moreover, IDR 428–473 with SLiM USP7, which acts as a deubiquitination enzyme has four nsSNVs and IDRs 597–602, and 626–647 embedding series of SLiMs in the β-barrel 2 domain has three nsSNVs. There are several other nsSNVs that overlap with IDR-associated SLiMs (Table 2), which might interfere with protein interactions and have possible functional consequences. Presence of heterozygous damaging nsSNVs within the LIR motifs of TGM2 may be also interesting to study since TGM2 is reported to play a role in autophagosome maturation [53] and it also interacts with autophagy cargo protein p62 to remove the ubiquitinated proteins [54].

Recent studies have identified core genes (~10% of the ~20,000 genes) essential for life in human cells in a context dependent manner [13, 14, 15]. These genes are highly conserved, do not duplicate during evolution, code for abundant proteins with multiple interactions in cells and show less than average polymorphism and deleterious mutations indicating strong purifying selection. Based on similar features one may consider TGM2 (and perhaps TGM7) also as an essential gene in humans; it is highly abundant in some cells (e.g. endothelial and smooth muscle cells) [1, 5], has broad interaction potential [4] and as shown above displays low polymorphism in the population scale. However, none of the transglutaminases are listed [13, 14, 15] as essential for at least proliferation and maintaining of immortalized human cell lines in culture. Much more population genomic data need to be generated and analyzed to decide whether there are TGM2 functions which are essential for the existence of the whole organism as implied by the data discussed here. It is still possible that disease causing very rare TGM2 variants will be found as full genome or exon sequencing becomes even more widely accessible. Studying these cases and continuation of systematic biochemical and *in vivo* investigations will be needed to answer the question what TGM2 functions reported and proposed so far are critical for human physiology.

## Supporting information

S1 Table Sequence and structure based predictions for single mutant human TGM2 proteins. Predicted probabilities of helices (H), extended strands (E), and coils (C) are displayed (%). Predicted secondary structure for wild-type is given in column 5 and for mutations in column 9. The stability values calculated for the opened and closed forms are shown in column 11 and 12. *Residue not present in the PDB file in closed form. Out of all studied damaging nsSNVs, only 8 nsSNVs mentioned in this table had minor effects on secondary structure and others did not influence the secondary structure.
(PDF)

S2 Table Transglutaminase heterozygous and homozygous LOF types from literature. Column 1 Transglutaminase genes; other columns represent different populations with number of homozygote individuals given in brackets except for column 5 (ARIC dataset) were heterozygote individuals are mentioned in brackets and 1 homozygote individual for TGM4. In case of ExAC dataset (column 6) only TGM4 and TGM6 had homozygote individuals and as there are no exact data available about heterozygote individuals, allele count is shown. Column 2 indicates Icelandic population study involving 104,220 individuals [10]; column 3 indicates study involving 3222 British-Pakistani-heritage adult individuals living in the UK [12]; column 4 denotes data from study involving 6970 individuals: (1496 cases and 5474 controls) sequenced to characterize rare complete knockouts in Autism spectrum disorders cases [59]; column 5 denotes data from study involving Atherosclerosis Risk in Communities [58]; and column 6 from ExAC datasets showing the allele count for F13a, TGM1-TGM7 and homozygote individuals for TGM4 and TGM6 [11].
(PDF)

S3 Table Transglutaminase mutations in OMIM database. The OMIM database was accessed on July 2016. Mutations are cataloged in OMIM in the allelic variants section of gene entries. Only selected examples listed in OMIM database is shown here. If a particular variant is present in homozygotes then it is mentioned in brackets. Full list of transglutaminase related references, are available in OMIM database.
(PDF)
