# Supplementary material for: Genomic variants reveal differential evolutionary constraints on human transglutaminases and point towards unrecognized significance of transglutaminase 2
Source: PLoS One. 2017 Mar 1;12(3):e0172189. doi: 10.1371/journal.pone.0172189 (PMC5332030; doi:10.1371/journal.pone.0172189)
Supplement: S1 Table — Predicted probabilities of helices (H), extended strands (E), and coils (C) are displayed (%). Predicted secondary structure for wild-type is given in column 5 and for mutations in column 9. The stability values calculated for the opened and closed forms are shown in column 11 and 12. *Residue not present in the PDB file in closed form. Out of all studied damaging nsSNVs, only 8 nsSNVs mentioned in this table had minor effects on secondary structure and others did not influence the secondary structure. (PDF) [file pone.0172189.s001.pdf]

S1 Table

	Sequence-based prediction									Structure-based prediction	
	Probability values for wild type residues (%)				Probability values for mutated residues (%)					Stability values (ΔΔG, kcal/mol)	
Mutation	H	E	C	sec. str.	H	E	C	sec. str.	sec. str. change	opened confor mation	closed confor mation
p.Leu12Pro*	60.8	16.6	22.6	H	38.4	3.6	58	C	H→ C	0.29	-
p.Arg214His	4.8	13.2	82.0	C	3.2	9.1	87.7	C	-	2.32	2.37
p.Val220Met	2.6	87.8	9.6	E	11.1	76.8	12.1	E	-	-0.17	-1.33
p.Val272Ile	0.4	81.0	18.6	E	0.4	82.5	17.1	E	-	-0.38	-0.29
p.Val283Met	3.0	40.0	56.9	C	4.5	23.9	71.6	C	-	5.46	0.13
p.Trp337Leu	0.4	90.1	9.5	E	0.2	94.2	5.6	E	-	1.74	2.25
p.Arg377His	14.6	53.7	31.7	E	9.3	47.0	43.7	E	-	0.84	1.35
p.Val431Leu	21.0	24.2	54.8	C	26.6	14.2	59.3	C	-	0.97	0.52
