# Supplementary material for: Genomic variants reveal differential evolutionary constraints on human transglutaminases and point towards unrecognized significance of transglutaminase 2
Source: PLoS One. 2017 Mar 1;12(3):e0172189. doi: 10.1371/journal.pone.0172189 (PMC5332030; doi:10.1371/journal.pone.0172189)
Supplement: S2 Table — Column 1 Transglutaminase genes; other columns represent different populations with number of homozygote individuals given in brackets except for column 5 (ARIC dataset) were heterozygote individuals are mentioned in brackets and 1 homozygote individual for TGM4. In case of ExAC dataset (column 6) only TGM4 and TGM6 had homozygote individuals and as there are no exact data available about heterozygote individuals, allele count is shown. Column 2 indicates Icelandic population study involving 104,220 individuals [10]; column 3 indicates study involving 3222 British-Pakistani-heritage adult individuals living in the UK [12]; column 4 denotes data from study involving 6970 individuals: (1496 cases and 5474 controls) sequenced to characterize rare complete knockouts in Autism spectrum disorders cases [59]; column 5 denotes data from study involving Atherosclerosis Risk in Communities [58]; and column 6 from ExAC datasets showing the allele count for F13a, TGM1-TGM7 and homozygote individuals for TGM4 and TGM6 [11]. (PDF) [file pone.0172189.s002.pdf]

S2 Table

Gene	Icelandic population	British Pakistani	Autism spectrum disorders cases	Atherosclerosis Risk in Communities (ARIC)	Exome Aggregation Consortium subjects (ExAC)
F13a	-	-	-	1 frameshift; 1 splicing; 1 stop gained (4 individuals)	7 stop gained; 4 splice donor; 3 splice acceptor; 8 frameshift Allele count: 86
TGM1	1 splice acceptor (2 individuals)	-	-	1 frameshift; 1 splicing; 2 stop gained (8 individuals)	10 stop gained; 2 splice donor; 3 splice acceptor; 4 frameshift; Allele count: 66
TGM2	-	-	-	2 stop gained (4 individuals)	13 stop gained; 2 splice donor; 6 splice acceptor; 8 frameshift; Allele count: 54
TGM3	1 stop gained	-	-	2 frameshift; 1 splicing; 1 stop gained (4 individuals)	7 stop gained; 1 splice donor; 3 splice acceptor; 9 frameshift Allele count: 27

TGM4	1 stop gained (9 individuals)	1 Stop gained	1 Stop gained (1 individual case)	2 frameshift; 1 splicing 5 stop gained (129 individuals; 1 homozygote)	14 stop gained; 10 splice donor; 0 splice acceptor; 15 frameshift; Allele count: 1179; (11 homozygotes)
TGM5	1 stop gained 1 splice acceptor (3 individuals)	-	-	3 frameshift; 1 splicing; 3 frameshift (8 individuals)	11 stop gained; 10 splice donor; 3 splice acceptor; 16 frameshift; Allele count: 85
TGM6	1 stop gained 1 splice acceptor	-	-	3 frameshift; 3 stop gained (11 individuals)	13 stop gained; 10 splice donor; 5 splice acceptor; 11 frameshift; Allele count: 237; (1 homozygote)
TGM7	-	-	-	2 frameshift; 3 splicing; 1 stop gained (21 individuals)	10 stop gained; 5 splice donor; 3 splice acceptor; 10 frameshift; Allele count: 74
