# Supplementary material for: Genomic variants reveal differential evolutionary constraints on human transglutaminases and point towards unrecognized significance of transglutaminase 2
Source: PLoS One. 2017 Mar 1;12(3):e0172189. doi: 10.1371/journal.pone.0172189 (PMC5332030; doi:10.1371/journal.pone.0172189)
Supplement: S3 Table — The OMIM database was accessed on July 2016. Mutations are cataloged in OMIM in the allelic variants section of gene entries. Only selected examples listed in OMIM database is shown here. If a particular variant is present in homozygotes then it is mentioned in brackets. Full list of transglutaminase related references, are available in OMIM database. (PDF) [file pone.0172189.s003.pdf]

S3 Table

F13a	TGM1		TGM5	TGM6
g.210_211del, (homozygous)	c.4640del (homozygous)	p.Asn288Thr	p.Gly113Cys (homozygous)	p.Leu517Trp
p.Arg681His (homozygous)	g.3366A>G (homozygous)	p.Arg306Trp	p.Lys445Asn (homozygous)	p.Asp327Gly
p.Arg171Ter	p.Ser42Tyr	p.Gly217Ser	p.Trp255Arg	p.Asp510His
p.Tyr441Ter	p.Arg142Cys	g.1303_1307del	p.Leu41Pro	p.Arg111Cys
p.Asn60Lys	p.Arg323Gln	p.Gln582Ter	c.640del	c.1722_1724del
p.Gly501Arg	p.Arg141His (homozygous)	g.86C>T	c.1811_1815delinsTCCTTCA	
p.Gly562Arg (homozygous)	p.Arg142His (homozygous)	p.Asp101Val		
p.Val414Phe (homozygous)	p.Val378Leu	p.Leu204Gln		
p.Arg260His (homozygous)	p.Arg395Leu	p.Tyr276Asn (homozygous)		
p.Val34Leu (homozygous)	p.Val382Met (homozygous)	p.Arg126Cys		
p.Arg326Gln	p.Arg388His	p.Arg315Cys		
p.Val316Phe	g.9008del	p.Arg315His (homozygous)		
p.Tyr283Cys	p.Asp490Gly	p.Arg315Leu (homozygous)		
c.1286_1287insC (homozygous)	p.Gly278Arg	p.Val359Met		
g.[5G>A;15C>T]	p.Gly392Asp	p.Arg396His		
p.Arg703Trp	p.Arg142Pro	g.1922_1928del		
p.Arg661Ter	p.Arg286Gln	g.1331_1332insA		
p.Met242Thr	p.Val518Met	p.Arg670Ter (homozygous)		
c.602_605del	p.Ser160Cys	g.1223_1227del		
n.1+12C>A	p.Gly94Asp			
